# Eisenmenger syndrome and endometrial carcinoma: a cardio-oncologic therapeutic dilemma: case report

**DOI:** 10.1093/ehjcr/ytag374

**Published:** 2026-05-22

**Authors:** Matheus Henrique Sicupira Lima, Ana Maria Thomaz, Isabella de Luna Kalil, Gustavo Foronda, Ana Cristina Sayuri Tanaka, Luís Henrique Wolff Gowdak, Maria do Patrocínio Tenório Nunes, Ana Carolina Buso Faccinetto

**Affiliations:** University of São Paulo Medical School, Av. Dr. Arnaldo, 455, Cerqueira César, São Paulo, SP 01246-903, Brazil; Department of Clinical Cardiology, Heart Institute, Hospital das Clínicas, USP. Av. Dr. Enéas Carvalho de Aguiar, 44, Cerqueira César, São Paulo, SP 05403-000, Brazil; Department of Clinical Cardiology, Heart Institute, Hospital das Clínicas, USP. Av. Dr. Enéas Carvalho de Aguiar, 44, Cerqueira César, São Paulo, SP 05403-000, Brazil; Department of Clinical Cardiology, Heart Institute, Hospital das Clínicas, USP. Av. Dr. Enéas Carvalho de Aguiar, 44, Cerqueira César, São Paulo, SP 05403-000, Brazil; Department of Clinical Cardiology, Heart Institute, Hospital das Clínicas, USP. Av. Dr. Enéas Carvalho de Aguiar, 44, Cerqueira César, São Paulo, SP 05403-000, Brazil; Department of Clinical Cardiology, Heart Institute, Hospital das Clínicas, USP. Av. Dr. Enéas Carvalho de Aguiar, 44, Cerqueira César, São Paulo, SP 05403-000, Brazil; Department of Internal Medicine, Central Institute, Hospital das Clínicas, USP. Av. Dr. Enéas Carvalho de Aguiar, 255, Cerqueira César, São Paulo, SP 05403-000, Brazil; Department of Clinical Cardiology, Heart Institute, Hospital das Clínicas, USP. Av. Dr. Enéas Carvalho de Aguiar, 44, Cerqueira César, São Paulo, SP 05403-000, Brazil

**Keywords:** Eisenmenger syndrome, Thromboembolic pulmonary hypertension, Endometrial carcinoma, Cardio-oncology, Uterine artery embolization, Case report

## Abstract

**Background:**

Eisenmenger syndrome (ES) represents the end stage of uncorrected congenital heart defects with long-standing left-to-right shunts that evolve into irreversible pulmonary vascular remodelling and pulmonary arterial hypertension. This report highlights the complex cardio-oncological management of a patient with ES and chronic thromboembolic pulmonary hypertension (CTEPH), who developed endometrial carcinoma complicated by life-threatening uterine bleeding.

**Case summary:**

A 66-year-old woman with an uncorrected *ostium secundum* atrial septal defect complicated by Eisenmenger physiology and CTEPH presented with vaginal bleeding. She was diagnosed with endometrioid adenocarcinoma. Due to prohibitive surgical risk, radiotherapy was selected as the primary treatment; however, bleeding persisted despite completion of 28 sessions. Interruption of warfarin therapy was contraindicated, given her high thrombotic risk. Following multidisciplinary team deliberation, uterine artery embolization (UAE) was performed successfully, achieving haemorrhage control without major haemodynamic instability. This minimally invasive intervention proved life-saving in a patient for whom both surgical and medical alternatives carried unacceptable risk.

**Discussion:**

The case exemplifies the intricate balance between anticoagulation and haemorrhagic control in ES complicated by malignancy. Severe hypoxaemia and haemostatic abnormalities inherent to ES amplify both bleeding and thrombotic risks, complicating oncological management. Tumour hypoxia secondary to chronic cyanosis may contribute to radioresistance and poor response to local therapy. The successful use of UAE underscores the vital role of a multidisciplinary cardio-gynaecology-oncology team in navigating life-threatening clinical conflicts and tailoring individualized strategies when conventional guidelines offer limited direction.

Learning points

- Eisenmenger syndrome patients present a paradoxical haemostatic state, predisposing them simultaneously to thrombosis and haemorrhage.
- Uterine artery embolization can be an effective and life-saving measure for controlling severe gynaecological bleeding in patients with prohibitive surgical risk and obligatory anticoagulation.

## Primary specialties involved other than cardiology

Paediatric Cardiology, Obstetrics and Gynaecology, Interventional Radiology.

## Introduction

Eisenmenger syndrome (ES) occurs when patients with significant congenital intra- and/or extracardiac left-to-right shunts develop irreversible pulmonary vascular injury and severely elevated pulmonary vascular resistance (PVR) in response to pulmonary overcirculation.^1–5^ When the PVR exceeds systemic vascular resistance (SVR), the shunt direction reverses to pulmonary-to-systemic or bidirectional, leading to cyanosis. This condition is usually accompanied by profound hypoxaemia, secondary erythrocytosis, thrombocytopenia, multi-organ involvement, and a prohibitive surgical risk.^4^ At this stage, closing the primary defect is contraindicated.^3^

The severe, long-standing pulmonary hypertension in ES can lead to massive dilation of the pulmonary artery trunk. In rare cases, this can cause life-threatening mechanical complications, such as the extrinsic compression of the left main coronary artery (LMCA).^6^ Furthermore, the *in situ* thrombosis and abnormal flow dynamics seen in ES create a high-risk environment for the development of chronic thromboembolic pulmonary hypertension (CTEPH), a distinct pathology that carries its own absolute indication for lifelong anticoagulation.^3,7^

In many developed nations, the absolute number of adults living with congenital heart disease (CHD) surpasses the number of children with the same conditions.^2^ As a result, adult care providers are becoming increasingly responsible for these patients, who now suffer from the same age-related diseases as their non-CHD counterparts, such as cancer.^1^ In this scenario, the role of a multidisciplinary approach to the patients with adult CHD cannot be overemphasized.

Cancer, a group of diseases typically associated with ageing, also requires a thorough multidisciplinary approach. As a prime example of an age-related malignancy, endometrial cancer frequently presents with hallmark symptoms such as postmenopausal bleeding and abnormal premenopausal bleeding.^8^ Its diagnosis is established by histopathology, and surgery is the primary treatment option for patients with endometrial carcinoma.^9^ Nevertheless, for patients with prohibitive surgical risk, radiation therapy alone can be used, though with worse outcomes, as noted by Bickenbach and colleagues, who found an 87% 5-year survival rate for patients with stage I carcinoma treated by surgery alone, compared with a 69% survival rate for those treated with radiation alone.^10^

Unmitigated bleeding is particularly problematic for patients with ES, who exhibit a paradoxical haemostatic profile, being susceptible to both thrombosis and haemorrhage.^4,5,11^ They are prone to thromboembolic events, a primary cause of mortality, while concurrently manifesting a bleeding diathesis. This diathesis is multifactorial, stemming from thrombocytopenia, thrombasthenia, as well as the depletion of von Willebrand factor and of various coagulation factors.^4^

As would be expected from this profile, navigating anticoagulation in a patient who needs it to avoid life-threatening complications but, at the same time, presents with active bleeding is an immense clinical challenge. This case demonstrates the critical role of a multidisciplinary team (MDT) in balancing a prohibitive surgical risk and the successful use of a non-standard, life-saving intervention. A video abstract summarizing this cardio-oncologic case is available as Supplementary material.

## Summary figure

**Figure:**
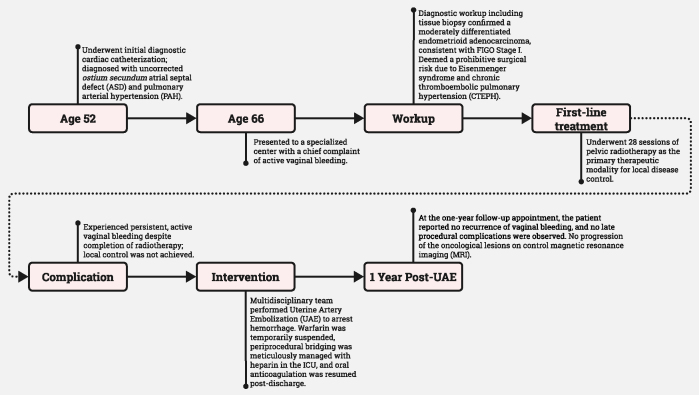


## Case presentation

A 66-year-old female patient, under follow-up at a specialized centre for an uncorrected *ostium secundum* atrial septal defect (ASD) complicated by ES, presented at one of her routine appointments complaining of vaginal bleeding. She was first diagnosed with an ASD at age 52, already with signs of pulmonary arterial hypertension (PAH). The patient was then referred to an obstetrician-gynaecologist, who diagnosed an endometrioid adenocarcinoma and requested a surgical risk assessment, given that the patient also lives with systemic arterial hypertension, major depressive disorder, and uterine leiomyomas. Additionally, she was under specialized monitoring due to extrinsic compression of the LMCA by the pulmonary artery trunk, which was markedly dilated as a result of ES. Her medication regimen at the time comprised warfarin 5 mg, enalapril 5 mg (twice daily), spironolactone 25 mg (once daily), ambrisentan 5 mg (once daily), atorvastatin 40 mg (once daily), and sertraline 50 mg (once daily).

The patient was in good general condition, eupnoeic, though exhibiting mild cyanosis. Vital signs were recorded: Blood pressure 100/60 mmHg, heart rate 70 bpm, SpO_2_ 94% on room air, in the seated position. Respiratory examination revealed symmetrical vesicular breath sounds with no adventitious sounds. Cardiac auscultation demonstrated a regular rhythm, featuring a Grade III/VI ejection systolic murmur and a Grade II/VI diastolic murmur over the mitral area. The patient displayed no jugular venous distention and had warm, well-perfused extremities without peripheral oedema. Before starting ambrisentan, her room-air oxygen saturation had been as low as 85%. On a subsequent 6-min walk test while on treatment, her oxygen saturation dropped from 94% to 90% after walking 350 m.

The patient's underlying diagnosis was an ASD, diagnosed and characterized via transthoracic echocardiography (TTE) (*Figure 1*), computed tomography pulmonary angiogram (CTPA) (*Figure 2*), and cardiac catheterization (*Figure 3*). TTE revealed a 28 mm *ostium secundum* ASD with bidirectional flow. Over time, this lesion led to progressive increases in PVR, resulting in elevated right heart chamber pressures and subsequent compensatory structural remodelling. Consistent with the progressive rise in PVR, TTE demonstrated signs of severe PAH, revealing a markedly elevated estimated right ventricular systolic pressure of 75 mmHg. This finding was confirmed by cardiac catheterization, which showed a mean pulmonary arterial pressure of 38 mmHg. TTE further revealed significant right heart remodelling, characterized by severe right ventricular hypertrophy and dilatation with mild systolic dysfunction secondary to apical hypokinesis.

**Figure 1:**
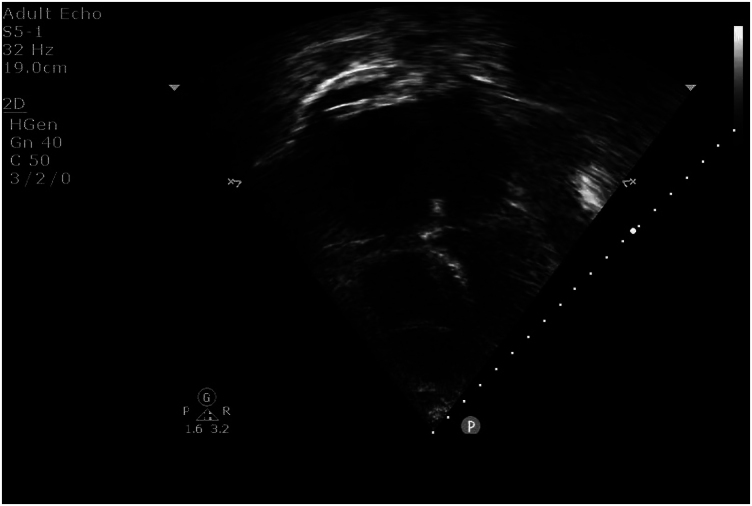
Transthoracic echocardiogram. Subcostal four-chamber view demonstrating a large *ostium secundum* atrial septal defect.

**Figure 2:**
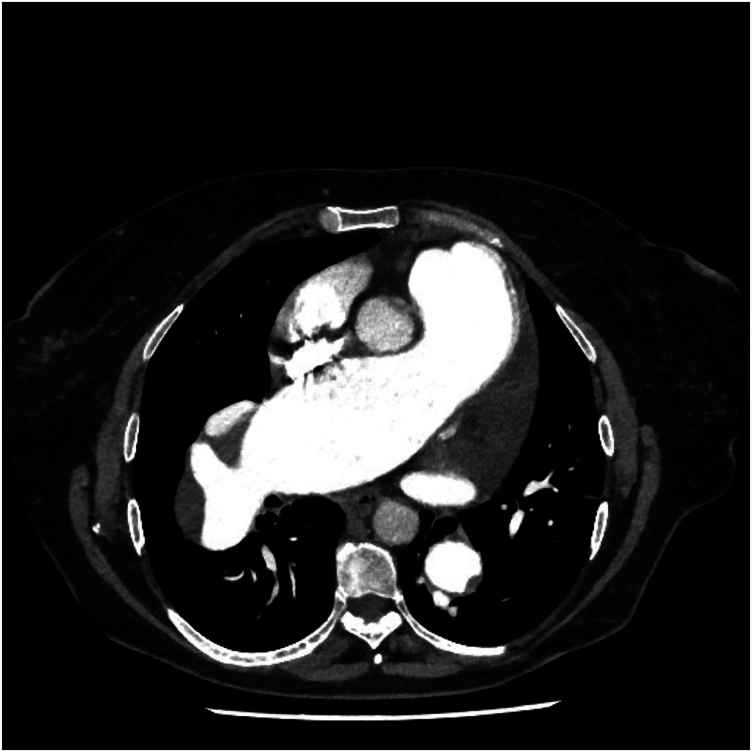
Axial computed tomography pulmonary angiogram. Contrast-enhanced axial image demonstrating aneurysmal dilation of the main pulmonary artery, which significantly exceeds the diameter of the adjacent ascending aorta. Notably, there is a large, eccentric, hypodense filling defect within the markedly dilated right pulmonary artery. In the clinical context of Eisenmenger syndrome, this is highly consistent with chronic *in situ* thrombosis rather than an acute embolic event.

**Figure 3:**
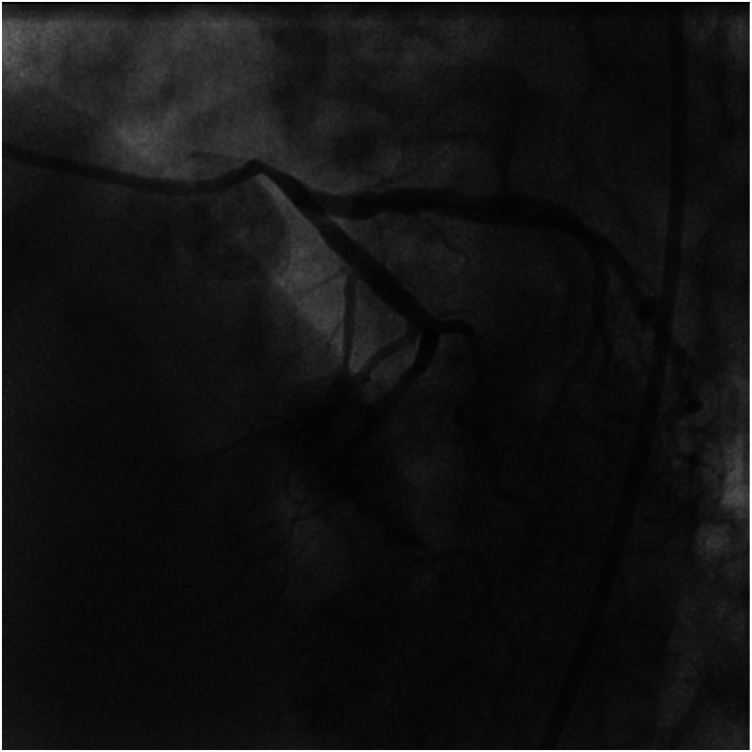
Selective left coronary angiography. Angiogram of the left coronary system demonstrating a 90% ostial stenosis caused by extrinsic compression from a markedly dilated pulmonary trunk.

Associated findings included severe tricuspid regurgitation and massive right atrial dilatation, visible on chest X-ray (*Figure 4*). The CTPA corroborated these findings, showing a markedly dilated pulmonary artery trunk (56 mm). Additionally, the CTPA also identified a partial, peripheral filling defect within the right pulmonary artery, specifically involving the proximal superior lobar and descending interlobar arteries, although segmental branches remained patent.

**Figure 4:**
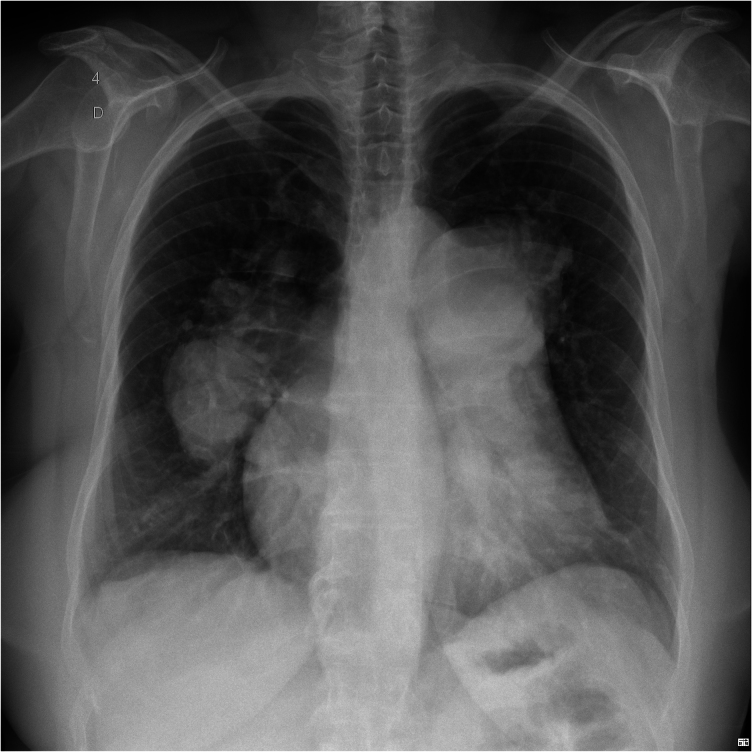
Posterior-anterior chest radiograph. The radiograph demonstrates gross cardiomegaly with right heart chamber enlargement. There is massive, aneurysmal dilation of the main pulmonary artery. Furthermore, extreme enlargement of the central right and left pulmonary arteries is noted at the hila, contrasting sharply with the rapid tapering of the peripheral pulmonary vasculature.

Cardiac catheterization identified a critical secondary finding: 90% ostial stenosis of the LMCA caused by extrinsic compression from the markedly dilated pulmonary trunk. LMCA compression is a recognized, albeit rare, complication of severe PAH, often presenting with exertional chest pain or ventricular arrhythmias. To assess the haemodynamic significance of this compression, a pharmacological stress myocardial perfusion scintigraphy with Gated-SPECT study was performed (*Figure 5*). The stress-induced ischaemia involved the mid-anterior (segment 7), apical anterior (segment 13), mid-anterolateral (segment 12), and apical lateral (segment 16) walls of the left ventricle. Given the prohibitive procedural risk associated with intervening on the extrinsic LMCA compression and the patient's asymptomatic state at the time, a conservative management approach was adopted; intervention was deferred as the potential benefit did not outweigh the significant perioperative risks.

**Figure 5:**
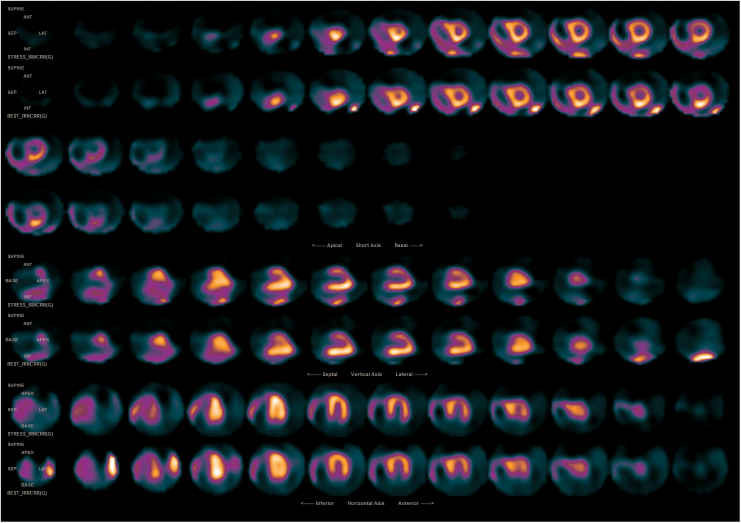
Pharmacological stress myocardial perfusion scintigraphy. Short-axis, vertical long-axis, and horizontal long-axis slices acquired during pharmacological stress and rest. The study demonstrates a reversible radiotracer perfusion defect, indicative of stress-induced ischaemia. Concurrently, there is strikingly intense radiotracer uptake within a severely dilated right ventricle.

The oncological workup included abdominal magnetic resonance imaging (MRI) (*Figure 6*), which revealed several incidental findings, followed by a tissue biopsy. The uterus was anteverted, anteflexed and enlarged, with lobulated contours attributed to multiple myometrial space-occupying lesions. These nodules exhibited hypoenhancement relative to the adjacent myometrium, consistent with uterine leiomyomas. Additionally, multiple hepatic nodules of varying sizes were detected, compatible with simple hepatic cysts. Histopathological analysis of the biopsy specimen revealed a moderately differentiated endometrioid adenocarcinoma.

**Figure 6:**
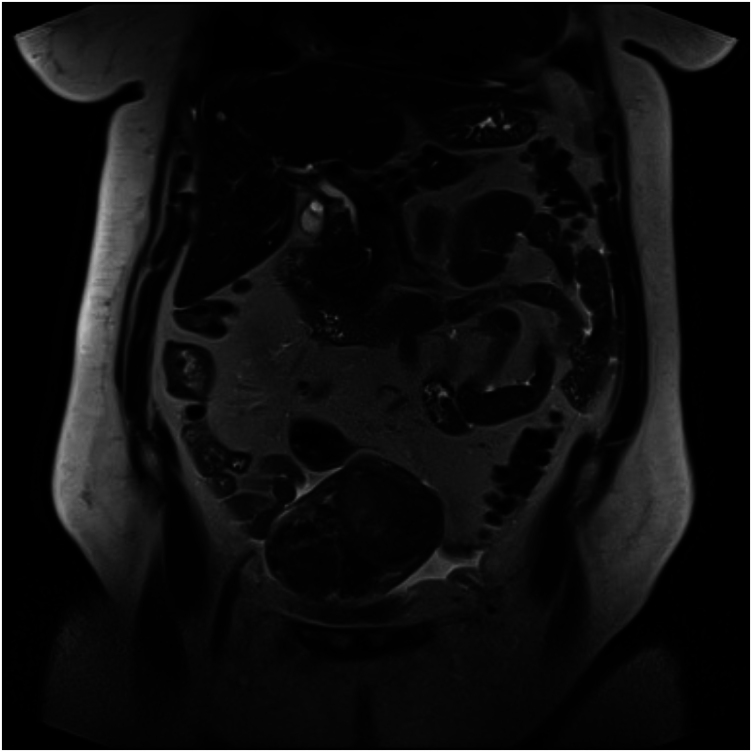
Coronal T2-weighted magnetic resonance imaging scan delineating an anteverted, anteflexed and enlarged uterus, with lobulated contours attributed to multiple myometrial space-occupying lesions. Hepatic lesions attributable to simple hepatic cysts were also seen.

Although endometrial carcinoma is typically managed surgically, operative intervention was contraindicated in this patient due to her prohibitive perioperative risk secondary to ES. Consequently, nonsurgical approaches were pursued. Pelvic radiotherapy was selected as the primary therapeutic modality for local disease control, a recognized alternative when definitive surgery is precluded. However, despite completing 28 sessions of radiation therapy, the patient unfortunately continued to experience active and persistent vaginal bleeding.

Following discussion with an MDT, the patient underwent uterine artery embolization (UAE) to address the persistent uterine bleeding. Warfarin was discontinued upon admission for the procedure and resumed immediately after discharge. The procedure was conducted under general anaesthesia, induced with etomidate. Airway and oxygenation management was achieved via invasive mechanical ventilation, and a multiparameter monitor was utilized. This included continuous electrocardiography, pulse oximetry, and non-invasive blood pressure monitoring on the right upper limb. The depth of anaesthesia was meticulously titrated using Bispectral Index monitoring to prevent anaesthetic overdose and subsequent cardiovascular depression. Fluid management included the administration of 500 ml of Ringer's lactate.

Vascular access was established via a puncture of the right common femoral artery utilizing a 5-French (5F) sheath. Initial assessment involved aortography performed with a 5F pigtail catheter, which successfully visualized both uterine arteries (*Figure 7*). These vessels were noted to be markedly enlarged and tortuous bilaterally. Subsequently, using a 5F Cobra II catheter and a 0.035-inch hydrophilic guidewire, selective catheterization of the internal iliac arteries was performed, followed by selective angiography of the uterine arteries and embolization with 500–700 and 700–900 μm microspheres. Following the successful procedure (*Figure 8*), the patient was immediately transferred to the CHD ICU for rigorous postoperative haemodynamic monitoring and stabilization.

**Figure 7:**
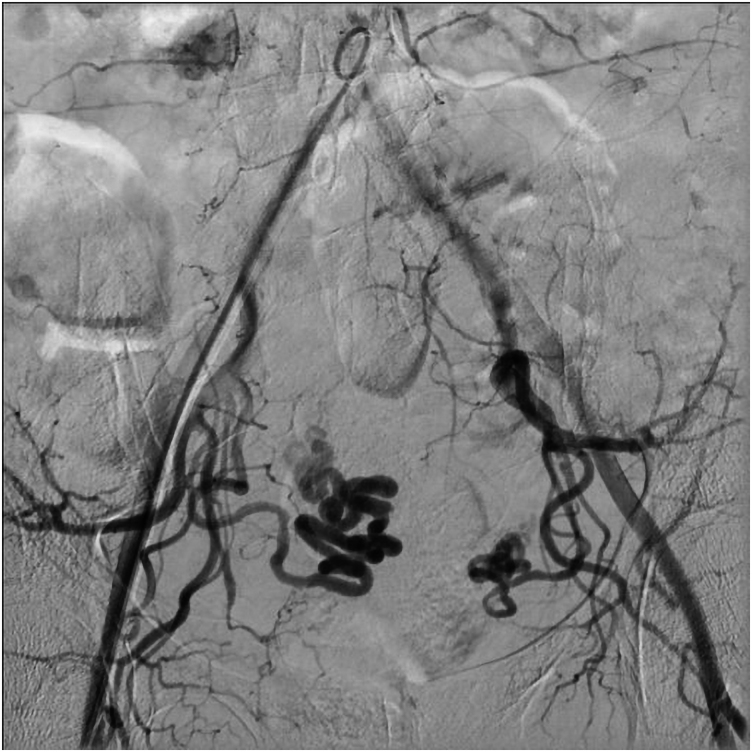
Digital subtraction angiography of the pelvis before uterine artery embolization. Pelvic angiogram demonstrating markedly hypertrophied and tortuous bilateral uterine arteries supplying a hypervascular network.

**Figure 8:**
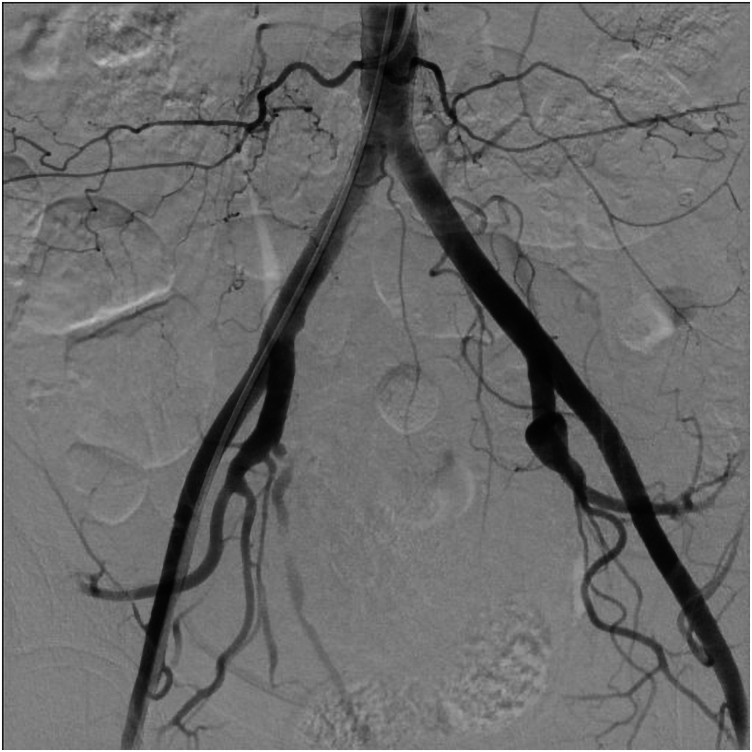
Digital subtraction angiography of the pelvis after uterine artery embolization. Completion angiogram following the targeted delivery of embolic agents. There is successful stasis of flow and complete devascularization of the target vascular territory, with preservation of normal antegrade flow in the main iliac arterial branches.

Continuous invasive haemodynamic monitoring was maintained in the ICU to promptly identify any drop in systemic blood pressure that could exacerbate the right-to-left shunt. During the acute postoperative phase, oral pulmonary vasodilator therapy was suspended to prevent systemic hypotension. Instead, inhaled nitric oxide (iNO) at 20 ppm was instituted to provide selective pulmonary vasodilation without affecting the systemic bed. Simultaneously, a continuous infusion of norepinephrine (0.07–0.15 μg/kg/min) was utilized to sustain SVR and prevent the worsening of the right-to-left shunt. However, during the weaning phase, the discontinuation of iNO and the reintroduction of oral pulmonary vasodilators precipitated acute systemic hypotension. In response, the team transiently reinstated the norepinephrine infusion as haemodynamic support. This swift intervention maintained SVR, facilitating the safe readaptation of the patient's cardiovascular system to her oral medications without further need for vasoactive or inhaled support.

Withheld during the periprocedural window, anticoagulation was reinitiated in the ICU using continuous intravenous unfractionated heparin. It was later transitioned to therapeutic low-molecular-weight heparin, strictly guided by anti-Xa levels rather than standard weight-based dosing. Once the anti-Xa target was achieved, a careful bridge to warfarin was started, and the patient was discharged.

At the 1-year follow-up appointment, the patient reported no recurrence of vaginal bleeding, and no late procedural complications were observed. Her endometrial cancer is currently being managed by a separate oncology team; as previously noted, formal surgical staging remains contraindicated due to her prohibitive surgical risk. However, her symptoms have not progressed to date, and there was no progression of the lesions in her follow-up MRIs.

## Discussion

This case epitomizes a profound diagnostic and therapeutic dilemma, stemming from the co-existence of ES secondary to a complex CHD and CTEPH. This confluence of high-risk pathologies, compounded by the management of a subsequent malignancy, created a significant cardio-oncology conflict. The scenario forced a critical re-evaluation of standard protocols, necessitating the precarious balancing of competing risks: specifically, the imperative to prevent catastrophic thromboembolic events, a risk actively elevated by ES and CTEPH, while mitigating the substantial threat of fatal haemorrhage induced by necessary antithrombotic therapy.

As previously noted, ES patients exhibit a paradoxical haemostatic profile, presenting susceptibility to both thrombosis and haemorrhage.^4,5,11^ Although routine anticoagulation is controversial in ES without additional risk factors,^3^ the risk of thrombosis is heightened by coagulation abnormalities, blood stasis in dilated cardiac chambers, atherosclerosis, and arrhythmias. In this patient, however, the diagnosis of CTEPH provided a definitive, co-existing indication. CTEPH mandates an aggressive, lifelong antithrombotic strategy using therapeutic doses of anticoagulation (Class I recommendation), typically with vitamin K antagonists such as warfarin, to prevent recurrent pulmonary thromboembolism, which is central to its pathophysiology.^12^

Consequently, the confluence of ES and CTEPH created multiple, severe, and intertwined indications for therapeutic anticoagulation.^4,5^ This scenario placed the patient at an exceedingly high risk of a lethal thrombotic event should therapy be interrupted.^3,13^ Conversely, the presence of malignancy introduced unique challenges that directly interacted with the existing cardiopulmonary pathology and established bleeding risk.^8^ Specifically, the baseline predisposition to haemorrhage associated with ES and cyanotic CHD was critically aggravated by the endometrial malignancy.

Although minor spontaneous bleeding (e.g. easy bruising or menorrhagia) is common and often self-limiting in patients with ES, major haemorrhage poses a substantial threat to life.^4^ Severe blood loss results in hypovolaemia and anaemia, which are extremely poorly tolerated in this population.^1–5^ ES patients maintain a precarious and fragile physiological equilibrium; any significant blood loss severely compromises this balance, driving the hypovolaemic deterioration that is a primary determinant of mortality in CHD and PAH patients during high-risk windows like the peripartum period or perioperative setting.^4^

The 2023 International Federation of Gynaecology and Obstetrics (FIGO) staging system for endometrial cancer states that the disease is surgically staged and pathologically examined.^14^ However, formal staging was precluded by prohibitive surgical risk. On magnetic resonance imaging, the biopsied lesion was confined to the uterine corpus, consistent with FIGO Stage I endometrial carcinoma. UAE is not used as a standard treatment for uterine carcinoma. Its primary role in gynaecologic oncology is limited to the management of intractable pelvic bleeding, such as palliative control of haemorrhage in patients with advanced or inoperable uterine malignancy, or as an adjunct to surgery in select cases where conventional haemostasis is not feasible.^15^ The Society of Interventional Radiology states that suspected uterine malignancy is an absolute contraindication to UAE, except when performed for palliation or adjunctive haemostasis.

As previously reported, the patient's severe cardiovascular risk profile mandated definitive radiotherapy as the primary management for her cancer, a strategy typically associated with favourable outcomes in non-operable, early-stage disease (up to 69% survival).^8^ However, this intervention had failed to achieve local control. This radioresistance is likely attributable to the patient's underlying cardiopulmonary status. Tumour hypoxia is a well-established driver of radioresistance, poor prognosis, and tumour aggressiveness in solid malignancies, including endometrial cancer.^16^ In this case, systemic hypoxaemia, a hallmark of ES, potentially exacerbated by underlying anaemia, likely compounded the tumour hypoxia by reducing oxygen delivery to the tumour microenvironment. Mechanistic data strongly support this link: low tissue oxygen tension promotes the upregulation of hypoxia-inducible factor-1 in endometrial cancer.^17^ These factors drive survival, angiogenesis, and therapy resistance. Although direct clinical evidence linking ES-related hypoxaemia to radiotherapy failure in this specific malignancy is lacking, the robust mechanistic and observational data make this relationship highly plausible.

Given the prohibitively high thrombotic risk associated with interrupting anticoagulation, the MDT selected an approach capable of arresting the haemorrhage without resorting to major abdominal surgery. In this complex clinical scenario, UAE was chosen as the optimal intervention to control life-threatening uterine bleeding. This minimally invasive procedure is a highly effective tool for rapid haemostasis and facilitates stabilization and recovery in patients who are not surgical candidates due to severe comorbidities, such as the patient's ES.^18^ Data regarding the long-term oncologic outcomes and survival impact of UAE in endometrial carcinoma remain sparse, reinforcing its utility as a life-saving measure rather than a curative one in this context.

## Data Availability

All data supporting the findings of this case report are available within the article.

## Lead author biography

Mr. Matheus Henrique Sicupira Lima is a medical student at the Faculty of Medicine, University of São Paulo, with primary clinical and research interests focusing on Internal Medicine and multimorbidity management.

## Notes

**Consent:** The authors confirm that informed consent for publication was obtained from the patient.
